# Subacute Limb Ischemia Suspected from COVID-19-Related Arterial Thrombosis Presenting with Local Occlusion Site Tenderness

**DOI:** 10.3400/avd.cr.23-00115

**Published:** 2024-04-10

**Authors:** Kenshiroh Kawabe, Masamitsu Suhara, Ryosuke Taniguchi, Yasuaki Mochizuki, Toshio Takayama, Katsuyuki Hoshina

**Affiliations:** 1 Division of Vascular Surgery, Department of Surgery, Graduate School of Medicine, The University of Tokyo, Tokyo, Japan; 2 Division of Vascular Surgery, International University of Health and Welfare Mita Hospital, Tokyo, Japan

**Keywords:** acute limb ischemia, COVID-19-related thrombosis, *in situ* thrombosis

## Abstract

A 59-year-old man was admitted to our hospital with severe popliteal fossa pain and mild left calf claudication. He had had an episode of pyrexia and dyspnea approximately 3 weeks prior. Contrast-enhanced computed tomography revealed acute occlusion of the left popliteal artery and multiple infiltration shadows with bilateral multifocal parenchymal consolidation of the lungs, suggesting post-coronavirus disease 2019 (COVID-19) pneumonia. As he had no comorbid risk of cardiogenic embolism or atherosclerosis, we diagnosed him with COVID-19-related arterial thrombosis. COVID-19-related arterial thrombosis should be considered a possible cause of acute limb ischemia, even when ischemic symptoms occur several weeks post infection.

## Introduction

Coronavirus disease 2019 (COVID-19) has been a focus of research, especially during the early phase of the COVID-19 pandemic, owing to the high incidence of acute thromboembolism.^1)^ While the incidence of fulminant COVID-19-related thrombosis decreased as later virus variants have emerged,^2)^ the precise pathological pathway or timing of occurrence remains unspecified.

Acute limb ischemia (ALI) requires urgent recanalization to prevent limb amputation and myonephropathic metabolic syndrome. The major etiology of ALI is an embolism of cardiac origin, such as atrial fibrillation or cardiac myxoma, with symptoms occurring suddenly and rapidly becoming severe. However, *in situ* thrombosis owing to an endothelial pathology, such as atherosclerosis, may develop more slowly and less severely.

Here, we describe the case of a patient with subacute limb ischemia owing to arterial occlusion without arrhythmia or atherosclerotic findings, which was exclusively diagnosed as COVID-19-related thrombosis.

## Case Report

In March 2022, a 59-year-old man was admitted to a nearby hospital with an 8-day history of mild left calf claudication after walking several hundred meters. Duplex ultrasonography revealed an occluded left popliteal artery and the patient was referred to our emergency department.

**Figure 1:**
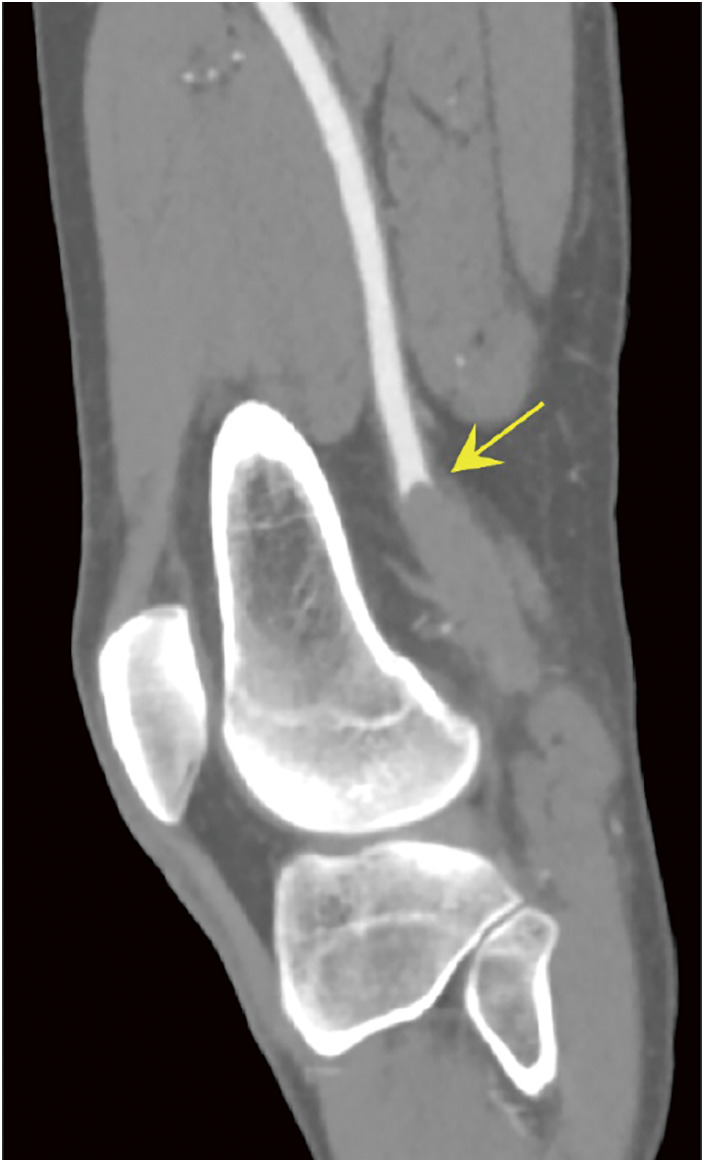
Fig. 1 Sagittal view of enhanced CT on initial presentation. The yellow arrow shows occlusion of popliteal artery. The top of thrombus is convex upward, which indicates acute occlusion. CT: computed tomography

**Figure 2:**
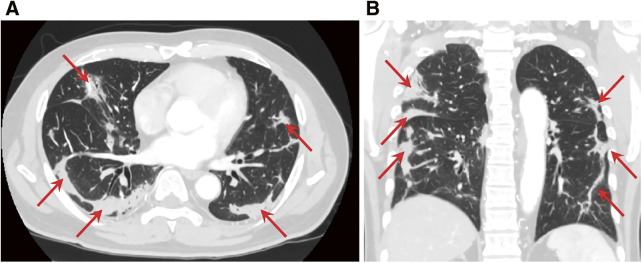
Fig. 2 Axial (**A**) and coronal (**B**) views of chest CT on first presentation. Red arrows show multiple areas of consolidation in peripheral regions of both lungs, which indicates post-COVID-19 pneumonia. COVID-19: coronavirus disease 2019

On physical examination, the left popliteal, posterior tibial, and dorsalis pedis pulses were nonpalpable. However, the left foot showed no pallor and the range of motion of his left foot joint was full. A manual muscle test and a sensory examination showed that it was so bilaterally. Thus, we considered that no neurological deficits were observed in the left foot. Severe tenderness was observed in the left popliteal fossa but not in the calf. Laboratory test results showed a slight elevation in the values of inflammatory markers, with a leukocyte count and C-reactive protein level of 10200/µL and 2.29 mg/dL, respectively. The D-dimer level was also elevated at 9.6 µg/mL. Muscle-derived serum enzyme levels, including the creatine kinase levels, were normal. Electrocardiography revealed sinus tachycardia. Contrast-enhanced computed tomography (CT) revealed proximal left popliteal artery occlusion; however, no remarkable atherosclerotic changes such as calcification and plaque were observed (**Fig. 1**). The upward convex shape of the pooled contrast medium at the occlusion site was typical of acute arterial occlusion. Almost no atherosclerotic changes were observed. Chest CT showed multiple areas of consolidation, with multifocal parenchymal consolidation in the peripheral region of both lungs, indicating a healed state following COVID-19-related pneumonia (**Figs. 2A** and **2B**).

Additional anamnesis revealed that the patient had a history of pyrexia and dyspnea, presented 3 weeks prior to admission. As he had not been to hospital, the precise cause was unknown. However, March 2022 was in the midst of the largest wave of the COVID-19 pandemic at the time, called “the sixth wave” in Japan. Severe acute respiratory syndrome coronavirus 2 (SARS-CoV-2) Omicron (BA.1/BA.2) was the dominant variant in the “sixth wave.” Considering the typical clinical symptoms and radiological findings after lung CT scan, the preceding respiratory symptoms were strongly suspected to be induced by COVID-19 pneumonia.

**Figure 3:**
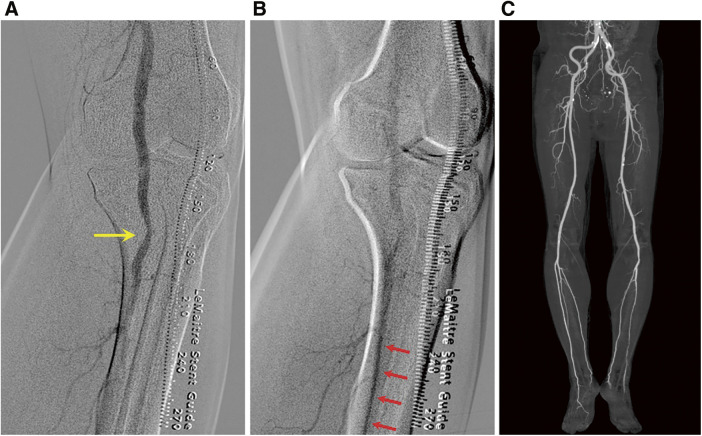
Fig. 3 Angiographic images after thrombectomy. (**A**) The popliteal artery displayed mild stenosis due to spasm (yellow arrow). (**B**) The peroneal artery exhibited good contrast (red arrows). (**C**) The absence of residual stenosis around the popliteal artery.

Thus, our findings revealed that arterial occlusion was caused by a COVID-19-related hypercoagulative state. As the polymerase chain reaction (PCR) test to rule out COVID-19 could not be performed during the holidays, emergent surgery was postponed due to the risk of infection. Fortunately, limb ischemia did not require urgent revascularization; the patient received heparinization, but the ischemia showed no improvement. PCR tests over consecutive days returned negative for COVID-19. We performed a thrombectomy via the left femoral artery 4 days following systemic anticoagulation therapy. Fresh thrombi were extracted using a 4-Fr Fogarty catheter. Postoperative angiography revealed effective revascularization of the infrapopliteal arteries. Although mild stenosis persisted in the popliteal artery, likely due to spasm (**Figs. 3A** and **3B**), we concluded that no further intraarterial administration of vasodilating agents was needed. We confirmed the pulse of left popliteal, posterior tibial, and dorsalis pedis artery after thrombectomy. Continuous intravenous administration of unfractionated heparin was maintained overnight.

One day post surgery, we switched from heparin to clopidogrel sulfate. The ankle–brachial index improved to 1.12. Postoperative magnetic resonance imaging revealed no adventitial cysts in the popliteal artery or popliteal entrapment syndrome. Contrast-enhanced CT indicated an improvement of the mild stenosis seen intraoperatively. We confirmed that the lesion had been a spasm and not a preexisting stenosis due to atherosclerosis, retrospectively (**Fig. 3C**). There were no notable pathological findings, such as tumor thrombus, cultural findings of the removed thrombi, nor serological findings suggestive of congenital coagulation deficiency. At 20 months after treatment, no recurrence of thrombosis was observed.

## Discussion

ALI etiology can be categorized as follows: thrombosis, embolism, and trauma. A recent study reported that 46% of all ALI cases were caused by arterial embolism and that 24% were caused by *in situ* thrombosis.^2)^ Embolism and trauma tend to induce more severe symptoms than thrombosis, given the drastic reduction in peripheral blood flow. Conversely, this patient had mild symptoms of intermittent claudication without the typical physical findings of ALI, namely, pain, pallor, pulse deficit, perishing cold, paresthesia, and paralysis, and slower development of ischemia (claudication lasting >1 week), despite a typical CT image of acute arterial occlusion. The clinical course of ALI is occasionally mild and slowly progressive in patients with atherosclerotic arterial changes. Preexisting stenotic lesions and subsequent collateral vessel development are assumed to attenuate the clinical symptoms of ALI. However, this patient had few atherosclerotic findings, including no arterial wall calcification on a CT scan and no comorbid risk factors such as dyslipidemia.

After confirming the typical findings of COVID-19-related pneumonia on a CT scan, we suspected COVID-19-related thrombosis and no embolism. We considered that this diagnosis might explain the discrepancy between the clinical course and radiological CT findings.

SARS-CoV-2, the causative virus in COVID-19, infects alveolar epithelial cells, which cause respiratory symptoms, and infects vascular epithelial cells via the angiotensin-converting enzyme 2 receptor.^3)^ This causes endothelial injury, produces a cytokine storm, and activates the coagulation system, leading to thrombi formation,^4)^ which can develop in veins and arteries throughout the body.^5)^

A high morbidity rate of venous thromboembolism in patients with COVID-19 has previously been reported. *In situ* pulmonary thrombosis has sometimes been observed,^6)^ possibly as a result of intimal cell deficiency via SARS-CoV-2 infection. This mechanism of viral infection via thromboangitis might explain local tenderness in the popliteal fossa. This atypical symptom as ALI might be a characteristic symptom to diagnose a COVID-19-related arterial thrombosis, paradoxically.

Another atypical threatening characteristic of COVID-19-related thrombosis is the high morbidity associated with arterial thrombosis. A systematic review reported that, among patients in the intensive care unit, the incidence of arterial thrombosis was 4.4%, with the extremities being the most common site of onset (39%).^7)^ One study reported that the estimated interval period before the onset of COVID-19-related arterial thrombosis was a median of 11 (5–20) days.^8)^ The subacute clinical course of ischemic pain and time interval between SARS-CoV-2 infection and the onset of pain in our patient appeared to fulfil the criteria of COVID-19-related thrombosis.

In the pre-COVID-19 era, ALI was reported to occur in 1–1.5 cases per 10000 persons annually^1^^,^^9)^; however, some studies have reported that during the COVID-19 pandemic, the ALI frequency increased. Bellosta et al. reported over five times as many patients with ALI in 2020 compared with those in 2019.^10)^ COVID-19-related arterial thrombosis was reported in only 4%–5% of patients with severe COVID-19 hospitalized in the intensive care unit in the aforementioned systematic review. Therefore, the estimated number of patients with COVID-19-related arterial thrombosis had increased to approximately 12 cases per 10000 persons annually. We consider that this five-fold increase in patients with ALI is plausible, despite the fact that the ratio of arterial thrombosis in all patients with COVID-19—not only those in intensive care units—would be lower and that not all occurrences of arterial thrombosis are present with ALI. We assume that there might have been more patients with undiagnosed COVID-19 and arterial thrombosis than was expected. COVID-19-related thrombosis should be considered as a possible cause of ALI in this pandemic, and a precise diagnosis might assist in determining appropriate timing for surgical treatment.

## Conclusion

In this study, we reported a case of subacute limb ischemia due to suspected COVID-19-related thrombosis. The discrepancy between the radiologic image showing typical ALI and a mild clinical course might be explained by the mechanics of the SARS-CoV-2 infection that developed during *in situ* arterial thrombosis and caused local thromboangiitis.

## Consent for Publication

The patient provided written informed consent to publish the case details and relevant images.

## Availability of Data and Materials

Not applicable.

## Ethics Approval and Consent to Participate

This study was approved by the Ethical Review Board of the University of Tokyo (IRB No. 3316-(5)).

## Disclosure Statement

All authors have no conflict of interest.

## Author Contributions

Patient care: all authors

Writing: KK and MS

Critical review and revision: all authors

Final approval of the article: all authors

Accountability for all aspects of the work: all authors.

## References

[1] Law N, Chan J, Kelly C, et al. Incidence of pulmonary embolism in COVID-19 infection in the ED: ancestral, Delta, Omicron variants and vaccines. Emerg Radiol 2022; 29: 625–9. 35446000 10.1007/s10140-022-02039-z PMC9022402

[2] Howard DPJ, Banerjee A, Fairhead JF, et al. Population-based study of incidence, risk factors, outcome, and prognosis of ischemic peripheral arterial events: implications for prevention. Circulation 2015; 132: 1805–15. 26350058 10.1161/CIRCULATIONAHA.115.016424 PMC4633967

[3] Bourgonje AR, Abdulle AE, Timens W, et al. Angiotensin-converting enzyme 2 (ACE2), SARS-CoV-2 and the pathophysiology of coronavirus disease 2019 (COVID-19). J Pathol 2020; 251: 228–48. 32418199 10.1002/path.5471 PMC7276767

[4] Thomas MR, Scully M. Clinical features of thrombosis and bleeding in COVID-19. Blood 2022; 140: 184–95. 35452509 10.1182/blood.2021012247 PMC9040438

[5] Jenner WJ, Kanji R, Mirsadraee S, et al. Thrombotic complications in 2928 patients with COVID-19 treated in intensive care: a systematic review. J Thromb Thrombolysis 2021; 51: 595–607. 33586113 10.1007/s11239-021-02394-7 PMC7882250

[6] van Dam LF, Kroft LJM, van der Wal LI, et al. Clinical and computed tomography characteristics of COVID-19 associated acute pulmonary embolism: a different phenotype of thrombotic disease? Thromb Res 2020; 193: 86–9. 32531548 10.1016/j.thromres.2020.06.010 PMC7274953

[7] Cheruiyot I, Kipkorir V, Ngure B, et al. Arterial thrombosis in coronavirus disease 2019 patients: a rapid systematic review. Ann Vasc Surg 2021; 70: 273–81. 32866574 10.1016/j.avsg.2020.08.087 PMC7453204

[8] Fournier M, Faille D, Dossier A, et al. Arterial thrombotic events in adult inpatients with COVID-19. Mayo Clin Proc 2021; 96: 295–303. 33549252 10.1016/j.mayocp.2020.11.018 PMC7691140

[9] Creager MA, Kaufman JA, Conte MS. Clinical practice. Acute limb ischemia. N Engl J Med 2012; 366: 2198–206. 22670905 10.1056/NEJMcp1006054

[10] Bellosta R, Luzzani L, Natalini G, et al. Acute limb ischemia in patients with COVID-19 pneumonia. J Vasc Surg 2020; 72: 1864–72. 32360679 10.1016/j.jvs.2020.04.483 PMC7188654

